# Discovery and implementation of transcriptional biomarkers of synthetic LXR agonists in peripheral blood cells

**DOI:** 10.1186/1479-5876-6-59

**Published:** 2008-10-16

**Authors:** Elizabeth A DiBlasio-Smith, Maya Arai, Elaine M Quinet, Mark J Evans, Tad Kornaga, Michael D Basso, Liang Chen, Irene Feingold, Anita R Halpern, Qiang-Yuan Liu, Ponnal Nambi, Dawn Savio, Shuguang Wang, William M Mounts, Jennifer A Isler, Anna M Slager, Michael E Burczynski, Andrew J Dorner, Edward R LaVallie

**Affiliations:** 1 Department of Biological Technologies, Wyeth Research, 35 CambridgePark Drive, Cambridge, MA 02140, USA; 2 Department of Cardiovascular and Metabolic Disease, Wyeth Research, 500 Arcola Road, Collegeville, PA 19426, USA; 3 Department of Drug Safety and Metabolism, Wyeth Research, 500 Arcola Road, Collegeville, PA 19426, USA; 4 Department of Clinical Translational Medicine, Wyeth Research, 500 Arcola Road, Collegeville, PA 19426, USA

## Abstract

**Background:**

LXRs (Liver X Receptor α and β) are nuclear receptors that act as ligand-activated transcription factors. LXR activation causes upregulation of genes involved in reverse cholesterol transport (RCT), including ABCA1 and ABCG1 transporters, in macrophage and intestine. Anti-atherosclerotic effects of synthetic LXR agonists in murine models suggest clinical utility for such compounds.

**Objective:**

Blood markers of LXR agonist exposure/activity were sought to support clinical development of novel synthetic LXR modulators.

**Methods:**

Transcript levels of LXR target genes ABCA1 and ABCG1 were measured using quantitative reverse transcriptase/polymerase chain reaction assays (qRT-PCR) in peripheral blood from mice and rats (following a single oral dose) and monkeys (following 7 daily oral doses) of synthetic LXR agonists. LXRα, LXRβ, ABCA1, and ABCG1 mRNA were measured by qRT-PCR in human peripheral blood mononuclear cells (PBMC), monocytes, T- and B-cells treated *ex vivo *with WAY-252623 (LXR-623), and protein levels in human PBMC were measured by Western blotting. ABCA1/G1 transcript levels in whole-blood RNA were measured using analytically validated assays in human subjects participating in a Phase 1 SAD (Single Ascending Dose) clinical study of LXR-623.

**Results:**

A single oral dose of LXR agonists induced ABCA1 and ABCG1 transcription in rodent peripheral blood in a dose- and time-dependent manner. Induction of gene expression in rat peripheral blood correlated with spleen expression, suggesting LXR gene regulation in blood has the potential to function as a marker of tissue gene regulation. Transcriptional response to LXR agonist was confirmed in primates, where peripheral blood ABCA1 and ABCG1 levels increased in a dose-dependent manner following oral treatment with LXR-623. Human PBMC, monocytes, T- and B cells all expressed both LXRα and LXRβ, and all cell types significantly increased ABCA1 and ABCG1 expression upon *ex vivo *LXR-623 treatment. Peripheral blood from a representative human subject receiving a single oral dose of LXR-623 showed significant time-dependent increases in ABCA1 and ABCG1 transcription.

**Conclusion:**

Peripheral blood cells express LXRα and LXRβ, and respond to LXR agonist treatment by time- and dose-dependently inducing LXR target genes. Transcript levels of LXR target genes in peripheral blood are relevant and useful biological indicators for clinical development of synthetic LXR modulators.

## Background

The liver X receptors (LXRα and LXRβ, also known as NR1H3 and NR1H2, respectively) belong to the nuclear hormone receptor family of ligand-activated transcription factors. LXRs are involved in controlling the expression of a spectrum of genes that regulate cholesterol biosynthesis and export in the liver as well as cholesterol efflux from peripheral tissues [1-3]. In this way, LXRs act as cholesterol sensors in the body. As such, the naturally occurring, activating ligands for LXRs *in vivo *include specific oxidized cholesterol metabolites such as 24 (S),25-epoxycholesterol, 22 (R)-, 24 (S)-, and 27-hydroxycholesterol [4]. When these ligands bind to LXRs, they displace co-repressors and allow the ligand-bound LXR (which forms an obligate heterodimer with retinoid X receptor (RXR), the receptor for 9-*cis*-retinoic acid) to regulate the expression of target genes by binding to specific promoter response elements (LXREs) in target genes of LXR action [5-8]. In the liver, LXRs regulate the expression of genes that control cholesterol metabolism and homeostasis, such as cholesterol 7α-hydroxylase (in mice), which controls the cholesterol/bile acid synthetic pathway, and sterol regulatory element-binding protein-1c, a key transcription factor that regulates expression of genes important in fatty acid biosynthesis [9,10]. The role for each LXR isoform in these processes has been elucidated by studies of pan-LXRα/β agonists in LXRα KO mice [11,12]. LXRα and β have also been shown to be expressed in macrophage, where they play an important role in regulating cholesterol efflux from macrophage in atherosclerotic lesions [13-15]. In macrophage, LXR activation results in the induction of several genes. Among these induced genes are those encoding the ATP-binding cassette proteins, such as ABCA1 and ABCG1, which are plasma membrane-associated transport proteins that are responsible for mediating cholesterol efflux as the initial step of the "reverse cholesterol transport" (RCT) process thereby controlling cholesterol mobilization from lipid-laden macrophages [16,17]. This "effluxed" cholesterol is subsequently transferred to plasma acceptor proteins such as high-density lipoprotein (HDL), which then delivers excess cholesterol to the liver [17] for eventual excretion. The action of LXR activation in the liver stimulates bile acid production and excretion of this cholesterol. In addition, LXRs are expressed in the intestine where they limit dietary cholesterol uptake by regulating the expression of ABC family members ABCA1 and ABCG5/ABCG8 that reside on the apical surface of enterocytes and act as efflux pumps moving cholesterol out of absorptive cells into the intestinal lumen [18].

Since LXRs are important regulators of reverse cholesterol transport in macrophages, we and others have developed synthetic LXR agonists that have been shown to be capable of stimulating macrophages in atherosclerotic plaques to efflux the scavenged cholesterol and limiting plaque progression [19-23]. This attribute is of particular disease relevance because lipid accumulation in these cells, through the uptake of oxLDL/LDL, is believed to be of fundamental importance to the etiology and pathogenesis of atherogenesis and atherosclerosis and other chronic inflammatory diseases [24-28]. We have recently developed a novel LXR agonist LXR-623 that has been shown to be anti-atherogenic in mouse models of atherosclerosis (manuscript in preparation).

To assist in the clinical development of LXR-623, we sought to identify peripheral blood biomarkers of LXR agonist exposure and activity. Initial biomarker discovery experiments in rodents revealed that peripheral blood cells respond to orally dosed LXR-623 by substantially increasing the transcriptional level of ABCA1 and ABCG1 in a dose-dependent manner. These data were confirmed in primate studies, where it was shown that peripheral blood cell expression of ABCA1 and ABCG1 mRNA was significantly increased in a dose-dependent manner by LXR-623 following 7 days of dosing. These findings were extended to human cells by treating PBMC from normal human donors *ex vivo *with LXR-623, which showed that ABCA1 and ABCG1 expression was similarly regulated in human peripheral blood cells. Furthermore, despite the assumption that monocytes (the circulating macrophage-precursor cell type in PBMC) are the only LXR agonist-responsive cell type in PBMC, it was shown that T- and B-cells (in addition to monocytes) also express LXRα and LXRβ and respond to LXR agonist treatment by upregulating ABCA1 and ABCG1 gene expression. Based upon these findings, external standard based qRT-PCR assays were developed to measure copy numbers of ABCA1 and ABCG1 transcripts in whole blood cell RNA from human subjects in a Phase 1 SAD (Single Ascending Dose) clinical study of LXR-623. In a representative subject both ABCA1 and ABCG1 transcripts were rapidly upregulated with similar temporal profiles following a single dose of LXR-623. We conclude that the pharmacodynamic effects of synthetic LXR agonist compounds can be measured *in vivo *by monitoring the expression of selected LXR target genes in peripheral blood cells. This approach should prove useful for future clinical development of the present compound and other candidate LXR agonist compounds.

## Methods

### Materials

All cell culture reagents were obtained from Gibco-Invitrogen (Carlsbad, CA). LXR agonists T0901317 [N-(2,2,2,-trifluoro-ethyl)-N-[4-(2,2,2,-trifluoro-1-hydroxy-1-trifluoromethyl-ethyl)-phenyl]-benzenesulfonamide] [8,22] and GW3965 [3-(3-(2-chloro-3-trifluoromethylbenzyl-2,2 diphenylethylamino)propoxy)phenylacetic acid] [29] were prepared following standard chemical syntheses from published literature. LXR-623 was synthesized by the Wyeth Chemical and Screening Sciences group. Mouse Universal Reference Total RNA (catalog # 636657) and Human Universal Reference Total RNA (catalog # 636538) was purchased from Clontech (Mountain View, CA).

### Mouse blood collection and RNA isolation

Blood (~300 uL) obtained from C57/Bl6 mice treated with LXR-623 agonist compound was immediately mixed with 1.3 mL of RNAlater (Ambion, Austin, TX), and frozen at -80°C until further processing to RNA. RNA was isolated from the thawed samples using the RiboPure Blood Kit (Ambion #1928) following the manufacturer's protocol. Quantitation of total RNA samples was performed using an Eppendorf BioPhotometer 6131. RNA quality was assessed using an Agilent BioAnalyzer with the RNA Nano-chip (Agilent Technologies, Santa Clara, CA).

### Rat blood and tissue collection and RNA isolation

Male Long Evans rats (Charles River Labs) weighing approximately 300 g were administered a single gavage treatment of 1 ml 2% Tween 80/0.5% methylcellulose containing sufficient compound to deliver the indicated doses. At various times following dosing, the rats were anesthetized with isoflurane and peripheral blood was removed by cannulation of the abdominal aorta. Approximately 2.5 ml blood was collected into PAXgene Blood RNA Tubes (Qiagen, Valencia, CA; # 262115) and RNA was prepared according to the manufacturer's protocol. Spleens were removed and frozen in liquid nitrogen prior to processing for RNA isolation using the RNeasy Mini RNA Isolation Kit (Qiagen). Total RNA was quantified by RiboGreen (Invitrogen, Carlsbad, CA). For determination of drug levels, compounds were extracted from EDTA plasma into 1:1 acetonitrile:water and quantified by LC/MS/MS.

### Non-human primate blood collection and RNA isolation

Cynomolgus monkeys were treated for 7 days with LXR agonist LXR-623 at either 15 mg/kg/day or 50 mg/kg/day PO. Serum and whole blood samples were collected at predose (day 0) and following dosing on day 7. Whole blood (2.5 ml) was collected into PAXgene Blood RNA Tubes (Qiagen catalog # 262115), incubated overnight at room temperature, frozen on dry ice and stored at -80°C. Isolation of RNA from PAXgene tubes was performed according to the manufacturer's protocol. Quantitation of total RNA samples was performed using an Eppendorf BioPhotometer 6131 (Eppendorf, Hamburg, Germany). RNA quality was assessed using an Agilent BioAnalyzer with the RNA Nano-chip (Agilent).

### Human PBMC and purified blood cell collection and RNA isolation

Whole blood was collected in 8 mL CPT tubes (Becton-Dickinson, Franklin Lakes, NJ) from healthy donors and the CPT tubes were processed for the isolation of PMBCs according to the manufacturer's protocol. All PBMC preps from a single donor were pooled for cell counts and subsequent analysis. The cell number and cellular composition of each PBMC fraction was determined by Pentra C60+ automated cell counter (Horiba ABX, Montpelier, France). For *ex vivo *treatment with LXR agonist, the purified PBMC were resuspended in culture medium (RPMI + 10% fetal calf serum + 1% penicillin/streptomycin with 1% L-glutamine), transferred to 6-well (9.5 cm^2 ^each) tissue culture dishes at approximately 5 × 10^6 ^cells per well, and 2 uM LXR-623 or vehicle (DMSO) were added. After 18 hours of culture, RNA isolation and qPCR analysis for LXRα, LXRβ, ABCA1, ABCG1, and PLTP was performed. At time of harvest, conditioned media was removed and centrifuged at 450 × g for 5 minutes to pellet any cells that were not adherent. The adherent cells remaining on the plate were lysed by the addition of 1.2 ml RLT lysis buffer (Qiagen) containing 150 mM 2-mercaptoethanol (Sigma, St. Louis, MO) to the plate, the lysed cells were scraped from the plate with a cell lifter, and the lysed cells in RLT buffer were transferred to the cell pellet from the centrifuged conditioned media. The cell pellet was resuspended by vortexing, and the total cell lysate was used for RNA isolation using the RNeasy Mini RNA Isolation Kit (Qiagen). Quantitation of total RNA samples was performed using an Eppendorf BioPhotometer 6131; RNA yields averaged 4.5 ug total RNA per culture well. RNA quality was assessed using an Agilent BioAnalyzer with the RNA Nano-chip (Agilent).

Fresh human PBMC, T cells, B cells, and monocytes from normal human donors were purchased from AllCells (Emeryville, CA). Each cell set was derived from the same donor for comparison of response within a donor. The cells were cultured, treated, and harvested as described above for the PBMC cultures.

### Human whole blood collection and RNA isolation

ABCA1 and ABCG1 expression was evaluated in human clinical samples from a Wyeth-sponsored, single-center Phase 1 single ascending dose (SAD) clinical study (3201A1-100) of LXR-623 encompassing 40 healthy human subjects. Whole blood was collected into PAXgene tubes 2 hours prior to dosing and at time points of 2, 4, 12, 24, and 48 hours following oral administration of a single dose of LXR-623. RNA was purified from the PAXgene tubes as described above for the non-human primate samples. Sample RNA quality was assessed using an Agilent BioAnalyzer with the RNA Nano-chip (Agilent), using the RIN (RNA Integrity Number) algorithm [30] provided with the instrument software. For these samples, the mean RIN ranged from 4.1–8.8, with a mean RIN of 6.8.

### Preparation and purification of cDNA

Purified RNA was converted to cDNA for subsequent qRT-PCR using the High Capacity cDNA Archive Kit (Applied Biosystems, Foster City, CA; PN4322171), following the manufacturer's protocol. cDNA was subsequently purified from the reaction mix using the QIAquick PCR Purification kit (Qiagen PN28104) according to the instructions provided with the kit.

### Quantitative RT-PCR

All quantitative RT-PCR (qPCR, TaqMan^®^) reactions described below were run on an Applied Biosystems 7500 Real Time PCR System using the following cycling parameters: Step 1: 50°C, 2 minutes; Step 2: 95°C, 10 minutes; Step 3: 95°C, 15 seconds; Step 4: 60°C, 1 minute; repeat Steps 3 and 4, 39 more times. Amplification of transcripts for the genes of interest in each sample was compared to the same assay run on a "standard curve" consisting of a dilution series of cDNA prepared from RNA from an appropriate tissue source, unless otherwise noted. Standard curve cDNA concentrations were determined empirically so that the C_T _values for the input experimental samples fell within the experimental range of the respective standard curve for each transcript of interest. Input cDNA amounts were determined by titration experiments for each transcript. Amounts were chosen that best allowed for changes in C_T _due to experimental conditions while remaining on the standard curve. Data analysis was performed according to the Relative Standard Curve Method [31].

Quantitative RT-PCR on mouse RNA samples utilized the following assays from Applied Biosystems: ABCA1, Mm00442646_m1; ABCG1, Mm00437390_m1. The mouse GAPDH transcript was measured for each sample to normalize the amount of input RNA for each reaction, using the Applied Biosystems Rodent GAPDH Control Reagent Kit (# 4308313). Amplification of the genes in each sample was compared to the same assay run on a "standard curve" consisting of a dilution series of cDNA prepared from RNA from a mixture of mouse tissues (Mouse Universal RNA, Clontech # 636657).

Quantitative RT-PCR on rat RNA samples utilized the following oligonucleotide probe/primer sets: ABCA1, probe FAM-AGGATGTGGTGGAGCAGGCG and primers, forward 5'-GGGTGGCTTCGCCTACTTG-3' and reverse-5'-GACGCCCGTTTTCTTCTCAG-3'; ABCG1, probe FAM-TCACACATCGGGATCGGTCTC and primers, forward 5'-GTACTGACACACCTGCGAATCAC-3' and reverse-5'-TCGTTCCCAATCCCAAGGTA-3'. The rat GAPDH transcript was measured for each sample to normalize the amount of input RNA for each reaction, using the Applied Biosystems Rodent GAPDH Control Reagent Kit (# 4308313).

For measuring monkey transcripts, primate-specific primer and probe sets for ABCA1 and ABCG1 were designed with Primer Express Software (Applied Biosystems, Foster City, CA). The ABCG1 probe, FAM-CTGGTGACGAGAGGCTTCCTCAGTCC and primers, forward 5'-GGCAGAATTTAAAACTGCAACACA-3' and reverse-5'-GGTGCCTGGTACTAAGGAGCAA-3', were designed using Rhesus macaque nucleotide sequence (Genbank Accession # BV209042). Human ABCA1 TaqMan^® ^reagents, reported previously [32] were used for ABCA1 quantitation following their validation using total RNA from cynomolgus monkey liver (Biochain, Hayward, CA) and results were normalized to human 18S rRNA (Applied Biosystems Eukaryotic 18S rRNA Control Assay Hs99999901_s1) following validation of this 18S rRNA assay on monkey RNA.

For measuring human transcripts, the following quantitative RT-PCR assays were obtained from Applied Biosystems: ABCA1, Hs00194045_m1; ABCG1, Hs00245154_m1; PLTP, Hs00272126_m1. The human GAPDH transcript was measured for each sample to normalize the amount of input RNA for each reaction, using the Human GAPDH Control Reagent Kit (# 402869). Amplification of the genes in each sample was compared to the same assay run on a "standard curve" consisting of a dilution series of cDNA prepared from RNA from a mixture of human tissues (Human Universal RNA, Clontech # 636538).

Measurement of ABCA1 and ABCG1 transcripts in blood samples from the human clinical study of LXR-623 in healthy human subjects was performed using the same Applied Biosystems human TaqMan assays as described above (ABCA1, Hs00194045_m1; ABCG1, Hs00245154_m1; GAPDH, Endogenous Control Kit # 402869). However, an ''external standard'' approach was utilized, in which TaqMan data from each assay is compared to a standard curve generated with known quantities of pre-prepared transcript for each target. ABCA1, ABCG1 and GAPDH cDNAs in pXL5 cloning vectors were obtained from Origene (Rockville, MD). Pure synthetic standards for each transcript were prepared by *in vitro *transcription and purified. Transcripts were quantitated, diluted to 10^9 ^copies/mL, aliquoted and stored at -80°C until use. Data generated from samples were compared to standard curves utilizing these synthetic standards, quantitated and normalized in terms of number of target transcripts per 10^6 ^GAPDH molecules.

For human TaqMan assays, two-step RT-PCR reactions were performed using the TaqMan Gold RT-PCR Kit from Applied Biosystems (cat # N808-0233) according to the manufacturer's instructions. The kit includes TaqMan PCR Core Reagents (catalog # N808-0228), TaqMan Reverse Transcription Reagents (catalog # N808-0234) and TaqMan GAPDH Control Reagents (catalog # 402869). qPCR reactions were run on an Applied Biosystems 7500 Real Time PCR System using the following cycling parameters: Step 1: 50°C, 2 minutes; Step 2: 95°C, 10 minutes; Step 3: 95°C, 15 seconds; Step 4: 60°C, 1 minute; repeat Steps 3 and 4, 39 more times. Data analysis was performed according to the Relative Standard Curve Method [31].

### Microarray analysis of global gene expression

PBMC were purified from normal human donors (n = 4), and separately treated *ex vivo *as described above with either 2 uM LXR-623 or vehicle (0.1% DMSO) for 18 hours. RNA was purified as described above, and amplified and labeled using the Ovation Biotin Labeling and RNA Amplification System (NuGEN, San Carlos, CA). The labeled RNA was then used for microarray analysis using the GeneChip^® ^HG U133 2.0 Plus array (Affymetrix, Santa Clara, CA). Expression profiling was performed on the GeneChips^® ^as described previously [33]. Hybridization signal intensities of probe sets representing each gene were measured for individual samples in each cohort group (LXR-623 treated *vs*. vehicle), and an average signal intensity for that gene was then calculated and compared to the average signal values from the other cohort. Genes were judged to be changed significantly by treatment if the change in the mean hybridization signal intensity for the probe set(s) representing that gene were > 2 fold higher or lower in the treatment group than in the control group, with a p-value < 0.05 as determined by Student's *t *test.

### Analysis of protein expression by immunoblotting

PBMC was isolated from human blood collected in 8 ml CPT-citrate tubes (within an hour of collection), and plated onto 100 mm tissue culture dishes in RPMI containing 10% FBS, 2 uM L-glutamine and 50 IU/ml penicillin and 50 ug/ml streptomycin at a density of 10 million cells/plate. After allowing cells to settle for 90 minutes, the cells were treated with or without LXR agonists (2 uM) for 24 hours or 48 hours. Cells were lysed at the end of the incubation in 1 × Cell lysis buffer (Cell Signaling Technologies) containing Pefabloc SC (protease inhibitor) on ice for 10 minutes (500 ul/plate). Both adherent and non-adherent cells were collected. Equal volumes (16.25 ul) of cell lysate were loaded into each well of NuPAGE 4–12% Bis-Tris gels (Invitrogen), and Full-Range molecular weight markers (RPN800, GE Healthcare) were used to assess molecular weights. Separated proteins were electroblotted onto a nitrocellulose membrane (Invitrogen). The membranes were blocked in 5% Blot-QuickBlocker (Gbiosciences, St. Louis, MO) for one hour followed by washing in washing buffer (PBS, 0.1% Tween20). To determine the equivalence of protein loading between samples, actin protein in each sample was detected by Western blotting using an anti-actin antibody (Actin (1–19)-HRP, Santa Cruz, 1:2000). In addition, protein loading was assessed by staining the membrane with Ponceau S (Sigma). Duplicate membranes were blotted separately with anti-ABCA1 (Novus Biologicals, NB400-10555, 1:500), anti-ABCG1 (Abcam AB36969, 1:2500), or anti-LXRα (Novus NB300-612, 1:400). Unbound antibodies were removed by washing the membrane three times for 15 minutes each in washing buffer and were then incubated with secondary antibodies (anti-goat-HRP, Chemicon or anti-rabbit-HRP, NEF812001 Perkin Elmer 1:2000) for one hour followed by another three washes in the washing buffers as above. Proteins of interest were detected by chemiluminescence using ECL Western blotting detection reagents (Amersham). Correct bands were identified by molecular weight, and specificity was confirmed by comparing with a duplicate blot incubated with a different antibody.

## Results

### LXRs are expressed in peripheral blood mononuclear cells

**Figure 1:**
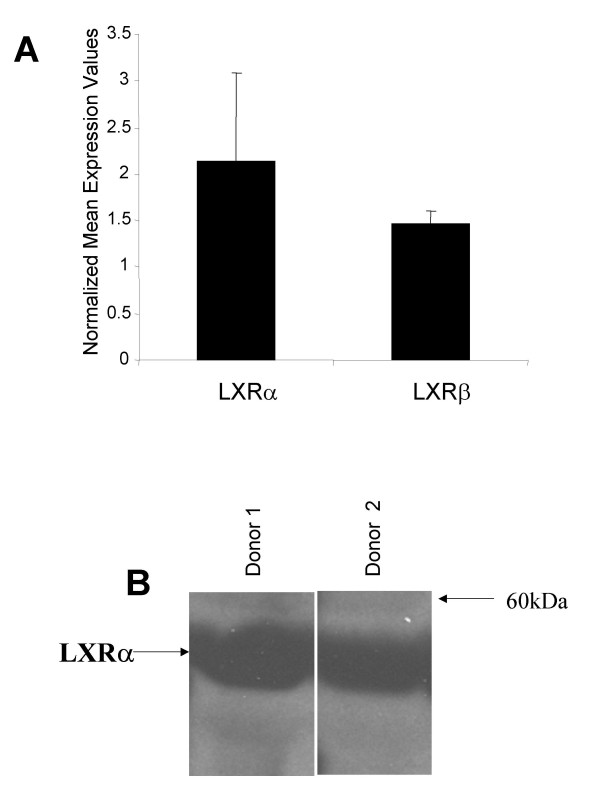
**LXRs are expressed in peripheral blood cells**. (A) RNA from peripheral blood mononuclear cells obtained from normal human donors was assayed for LXRα and LXRβ transcript levels using qPCR. Expression values were normalized to GAPDH levels, represented as the mean +/- SEM. (B) LXRα protein levels in protein extracts from PBMCs from these same donors were detected by Western blotting using rabbit anti-human LXRα polyclonal antisera.

Expression of LXRα and β in tissue macrophage and differentiated THP-1 cells has been well established [8,15,34-36], but scant evidence exists for expression of LXRs in circulating peripheral blood cells. Therefore, quantitative RT-PCR (TaqMan^®^) was performed on RNA isolated from PBMC from normal human donors, using assays designed to measure human LXRα or LXRβ transcripts. LXRα and LXRβ were both found to be expressed in PBMC (Fig. 1A). The presence of LXRα protein was confirmed by Western blotting of cell lysates from purified human PBMC from two separate donors with an anti-LXRα polyclonal antibody (Fig. 1B). Western analysis with LXRβ antisera in these same lysates was attempted but failed to detect a specific band of the proper size, possibly due to technical difficulties related to the available anti-LXRβ antibodies that were used (data not shown).

### LXR agonists induce gene expression in rodent peripheral blood cells *in vivo*

**Figure 2:**
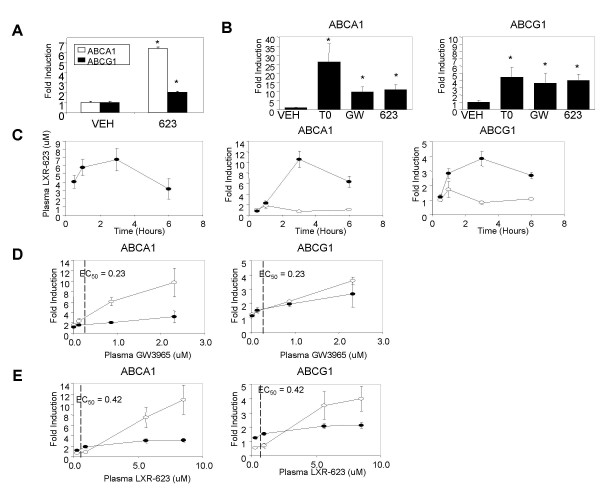
**LXR agonists increase ABCA1 and ABCG1 mRNA levels in rat peripheral blood cells**. (A) Normal C57/Bl6 mice on normal chow were orally dosed with a single administration of 50 mg/kg LXR-623 (623) or vehicle (VEH). At 4 hours post-dosing, peripheral blood expression of ABCA1 and ABCG1 mRNA was quantified by real-time PCR, using GAPDH as the normalizer. The bars indicate the normalized mean transcript levels +/- SEM (n = 4 per group). (B) Male Long Evans rats were administered a single dose of 10 mg/kg T0901317 (T0), 30 mg/kg GW3965 (GW), 30 mg/kg LXR-623 (623) or vehicle (VEH) by oral gavage. Three hours later peripheral blood expression of ABCA1 and ABCG1 mRNA was quantified by real-time PCR (100 ng RNA/assay). All expression values were normalized for GAPDH mRNA, with the level of expression in rats treated with vehicle defined as 1.0. Values are the mean +/- SEM (n = 6 per group). (C) Male Long Evans rats were administered a single dose of vehicle (open circles) or 30 mg/kg LXR-623 (filled circles) by oral gavage. At the indicated time points plasma concentration of LXR-623 (uM) and peripheral blood cell expression of ABCA1 and ABCG1 were determined. Values are the mean +/- SEM (n = 6 per group). (D) Male Long Evans rats were administered a range (0.01 to 30 mg/kg) of GW3965 by oral gavage. Three hours later plasma GW3965 concentration, peripheral blood ABCA1 and ABCG1 expression, and spleen ABCA1 and ABCG1 expression were quantified. The induction of gene expression in the peripheral blood (open circles) and spleen (filled circles) is plotted as a function of the plasma drug concentration. The EC50 for GW3965 induction of ABCA1 expression in murine J774 macrophages is denoted for reference. Values are the mean +/- SEM (n = 6 per group). (E) As above, except that rats were treated with a range (1 to 30 mg/kg) of LXR-623. * p < 0.01 compared to vehicle treatment, as determined by Student's *t *test.

To determine whether the presence of LXRα and LXRβ in peripheral blood cells would result in regulation of gene expression, a single oral dose of LXR-623 was administered to normal C57/Bl6 mice. Four hours post-dosing, the transcript levels of LXR target genes ABCA1 and ABCG1 in peripheral blood RNA were significantly increased compared to vehicle-treated mice (Figure 2A). A more comprehensive study was performed in rats, in which three structurally diverse LXR agonists, T0901317, GW3965, and LXR-623 were administered to normal male rats. Three hours following treatment, the expression levels of LXR target genes ABCA1 and ABCG1 were strongly induced in RNA from whole blood of all animals treated with the LXR agonists (Figure 2B). In both rodent species, the magnitude of ABCA1 induction was significantly greater than the magnitude of ABCG1 induction. In rats, the induction of ABCA1 and ABCG1 expression in peripheral blood cells was temporally correlated with plasma drug levels, with plasma concentrations of LXR-623 and ABCA1 and ABCG1 expression peaking three hours after a single dose (Figure 2C) and then diminishing as plasma drug levels decreased with clearance. Finally, to determine whether the *in vivo *elevation of ABCA1 and ABCG1 mRNAs reflected the potency of agonists to activate LXR receptors, rats were treated with a range of doses of GW3965 (Figure 2D) or LXR-623 (Figure 2E). Since the potency of these ligands for activation of rat LXRα or LXRβ is not known, the potency for activation of ABCA1 expression in mouse J774 macrophages (data not shown) was used as an approximation. For GW3965, significant induction of ABCA1 or ABCG1 in peripheral blood cells did not occur until plasma concentrations moderately exceeded the 0.23 uM EC50 for ABCA1 induction in J774 cells. Similarly, induction of ABCA1 and ABCG1 in peripheral blood cells by LXR-623 also required plasma concentrations in excess of the 0.42 uM EC50 for ABCA1 induction in J774 cells. Together, the dose dependence, temporal correlation, and activity of three structurally diverse ligands indicate that *in vivo *peripheral blood ABCA1 and ABCG1 gene expression is directly regulated by LXR.

Although gene induction in peripheral blood was correlated with plasma drug levels, the critical physiological effects of LXR activation are thought to reside within tissues such as the intestine, liver, or macrophages within the atherosclerotic lesion. Gene expression or drug concentration within these tissues cannot be easily monitored. To determine whether activation of gene expression in peripheral blood cells could provide insight into gene regulation within tissues, the induction of ABCA1 and ABCG1 within the spleen, an organ highly enriched in immune system cells, was compared to induction in peripheral blood cells. For GW3965, there was a strong correlation between the induction of ABCA1 or ABCG1 in the blood and spleen (Figure 2D). However, for LXR-623 the spleen appeared to have increased sensitivity relative to the peripheral blood at low plasma concentrations (Figure 2E). Whether this difference between ligands reflects differing levels of LXRα and LXRβ expression in blood cells *versus *spleen, or is due to some other factor such as differing coactivator abundance, remains to be determined. These initial results indicate that induction of LXR target gene regulation in the peripheral blood may serve as an indicator of target gene induction in relevant tissues.

### ABCA1 and ABCG1 transcription in peripheral blood cells of non-human primates is regulated in a dose-dependent manner by oral dosing of LXR-623

**Figure 3:**
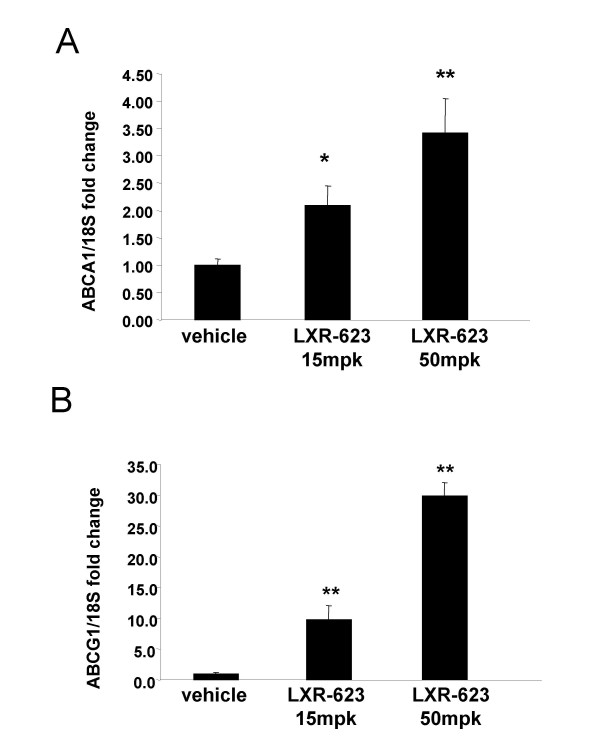
**LXR-623 upregulates transcription of ABCA1 and ABCG1 in monkey whole blood cells proportional to dose**. Cynomolgous monkeys maintained on normal chow were orally dosed with 0, 15 and 50 mpk/day of LXR-623 for 7 days (n = 4 per dose group). Blood was collected on day 7 of dosing, and RNA was prepared from whole blood for gene expression analysis of ABCA1 and ABCG1. qPCR was performed using assays designed to measure monkey (A) ABCA1 and (B) ABCG1 transcripts, and the measured amounts of these transcripts were normalized to monkey 18S RNA levels in each sample. Bars indicate the mean fold change of normalized ABCA1 or ABCG1 transcript levels +/- SEM in the indicated dose group compared to vehicle treated animals at the same time point. *p < 0.05, **p < 0.01 compared to vehicle treatment, as determined by Student's *t *test.

A study was performed in non-human primates to determine whether peripheral blood cells in higher species are responsive to LXR agonist treatment, and to evaluate the effect of prolonged LXR agonist dosing on peripheral blood expression of ABCA1 and ABCG1. Twelve cynomolgous monkeys maintained on normal chow were orally dosed with 0, 15 and 50 mg/kg/day of LXR-623 (n = 4 per dose group). Blood was collected prior to the first dose (day 0) to serve as a baseline and again on day 7. RNA prepared from whole blood was used for gene expression analysis of ABCA1 and ABCG1 by qPCR. In contrast to rodents, ABCG1 changed with much greater magnitude in primate blood cells than ABCA1 in response to LXR-623 at all doses tested (Figure 3). At day 7, ABCA1 expression (Figure 3A) was significantly increased by 15 mg/kg/day LXR-623 (2.1 fold *vs*. vehicle, p = 0.0135) and 50 mg/kg/day LXR-623 (3.4 fold *vs*. vehicle, p = 0.0006). The data suggested a dose-dependent increase in ABCA1 expression between the 15 mg/kg/day and 50 mg/kg/day doses at day 7, but the difference between doses did not reach significance (p = 0.12). Peripheral blood induction of ABCG1 by LXR-623 treatment at day 7 was much greater than was seen for ABCA1; the 15 mg/kg/day dose group showed levels of ABCG1 significantly increased by 9.8 fold *vs. *vehicle (p < 0.001) and dosing at 50 mg/kg/day increased ABCG1 levels by 29.8 fold *vs. *vehicle (p < 0.001). The difference between doses was also significant (p < 0.001).

### Human peripheral blood mononuclear cells respond to *ex vivo *LXR-623 exposure by increasing expression of LXR target genes

To determine whether the transcriptional effects of LXR agonists on peripheral blood cells that were seen in mouse and monkey could be translated to humans, PBMC were purified from normal human donors and treated in culture with either vehicle (0.1% DMSO), 0.05 uM or 2 uM LXR-623 for 18 hours. RNA purified from these PBMC cultures was profiled using Affymetrix HG U133 Plus 2.0 arrays to evaluate the genes that are regulated in peripheral blood cells by LXR-623. Table 1 shows a list of genes associated with reverse cholesterol transport and lipoprotein metabolism that were significantly changed in human PBMC by treatment with LXR-623. ABCA1 and ABCG1 were two of the top genes that changed with the greatest magnitude and significance. Other genes that have been previously shown to be regulated by LXR in various target tissues were found to be regulated in human PBMC by LXR-623, including steroyl-CoA desaturase [37], apolipoproteins C1 and C2 [38], phospholipid transfer protein [39], low density lipoprotein receptor [40], apolipoprotein E [38], and LXRα itself (NR1H3) [41].

**Table 1:** Up-Regulated Human Peripheral Blood Biomarkers of LXR-623 Activity

**Gene Symbol**	**Gene Title**	**Fold Change**	**p-Value**
ABCG1	ATP-binding cassette, sub-family G (WHITE), member 1	43.83	7.9E-08
SCD	stearoyl-CoA desaturase (delta-9-desaturase)	24.59	1.4E-07
ABCA1	ATP-binding cassette, sub-family A (ABC1), member 1	19.54	7.0E-08
APOC1	apolipoprotein C-I	13.37	2.5E-07
SREBF1	sterol regulatory element binding transcription factor 1	6.60	2.7E-03
PLTP	phospholipid transfer protein	5.81	9.0E-05
APOC2	apolipoprotein C-II	4.17	4.2E-06
LDLR	low density lipoprotein receptor (familial hypercholesterolemia)	3.91	2.4E-04
NR1H3	nuclear receptor subfamily 1, group H, member 3	3.85	1.0E-03
FADS1	fatty acid desaturase 1	3.01	3.9E-05
APOE	apolipoprotein E	2.85	1.6E-02

Selected genes changed significantly in human PBMC following *ex vivo *treatment with LXR-623. Peripheral blood mononuclear cells were purified from normal human donors (n = 4) and treated in culture with either vehicle (0.1% DMSO) or 2 uM LXR-623 for 18 hours. RNA purified from these PBMC cultures was profiled using Affymetrix HG U133 Plus 2.0 arrays to evaluate the genes that are regulated in peripheral blood cells by LXR-623. Shown is a list of genes associated with reverse cholesterol transport and lipoprotein metabolism that were significantly changed in human PBMC by treatment with LXR-623, along with fold-change and statistical significance.

**Figure 4:**
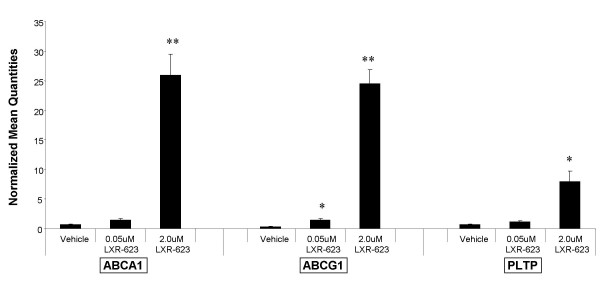
**LXR-623 treatment of human PBMC *in vitro *significantly increases transcription of ABCA1 and ABCG1**. Peripheral blood mononuclear cells (PBMC) were purified from normal human donors (n = 3), transferred to cell culture dishes, and treated with vehicle (0.1% DMSO) or LXR-623 at either 0.05 uM or 2 uM for 16 hours. Following culture, cells were harvested and RNA was isolated for gene expression measurements of human ABCA1, ABCG1, PLTP, and GAPDH (normalizer gene) using qPCR. Bars indicate the average normalized transcript level across the three donors for each dose, +/- SEM. *p < 0.05, **p < 0.01 compared to vehicle treatment, as determined by Student's *t *test.

**Figure 5:**
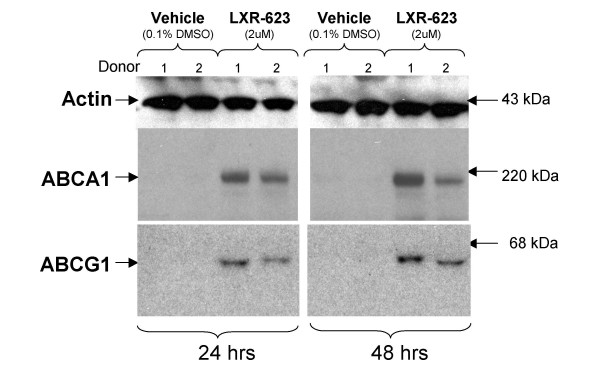
**LXR-623 treatment of human PBMC *ex vivo *significantly increases protein levels of ABCA1 and ABCG1**. Peripheral blood mononuclear cells (PBMC) were purified from normal human donors (n = 3), transferred to cell culture dishes, and treated with vehicle (0.1% DMSO) or LXR-623 (2 uM) for either 24 or 48 hours. Following incubation, cells were lysed and protein extracts were separated on SDS-PAGE and blotted with antisera raised to ABCA1, ABCG1, or actin (to serve as an indicator of protein loading per lane). Horseradish peroxidase-linked secondary antibodies were bound to the immobilized protein/antibody complexes, and proteins were visualized by chemiluminescence. Duplicate lanes for each treatment reflect the two different donors analyzed in this experiment. Molecular masses were estimated by the relative mobility of protein markers run in an adjacent lane on each gel.

The regulation of these target genes by LXR-623 in human PBMC was confirmed by a second set of experiments using blood from different human donors. qRT-PCR assays designed to measure human ABCA1, ABCG1, and PLTP were performed on RNA obtained from purified human PBMC treated in culture with LXR-623 as described above for the gene chip experiments. These experiments confirmed that mRNA for ABCA1, ABCG1, and PLTP was significantly upregulated in human PBMC by LXR-623 (Figure 4). In addition, this transcriptional induction was found to result in increased levels of ABCA1 and ABCG1 protein in the PBMC cell lysates as determined by Western blotting (Figure 5).

### Multiple cell types in human PBMC express functional LXRα and LXRβ

**Figure 6:**
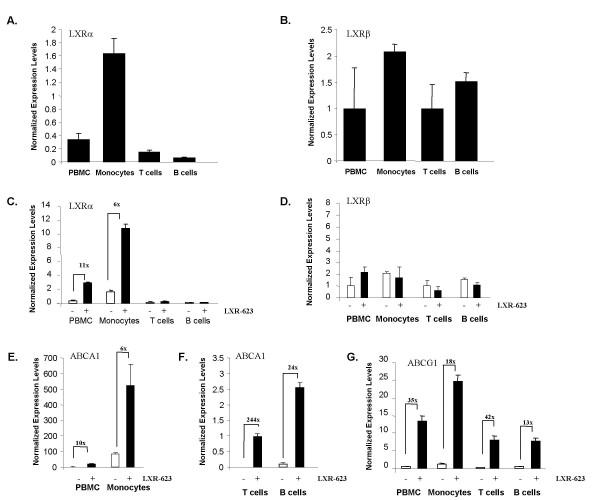
**All cell types in human PBMC express functional LXRα and LXRβ**. Peripheral blood mononuclear cells, monocytes, T-cells, and B-cells were purified from normal human donors (n = 3). After acclimation for 1 hour in culture, replicate cultures for each cell type from each donor were treated with either vehicle (0.1% DMSO) or LXR-623 (2 uM) for 18 hours. Cells were then harvested, RNA prepared, and qPCR was performed (in duplicate) to monitor the expression of LXRα, LXRβ, ABCA1, or ABCG1. Expression values were averaged across donor cultures for each treatment and normalized to GAPDH. A and B: mean basal levels of LXRα (A) and LXRβ (B) in vehicle-treated cultures after 18 hours in culture for each cell type. C-G: expression in vehicle treated (open bars) or LXR-623 treated cultures (black bars) of LXRα (C), LXRβ (D), ABCA1 (E, F), and ABCG1 (G). All bars represent the mean of replicate cultures from all donors +/- SEM. All fold-changes indicated in the graphs were significant with p < 0.01 by Student's *t *test.

Since it is well documented that macrophages express LXRs and respond to LXR agonists by increasing expression of certain LXR target genes [14,15,35], it was presumed that the LXR-responsive cell type in PBMC would most likely be monocytes, the precursor cell type to macrophages. To test this hypothesis, PBMC and the component cell-types of PBMC (moncytes, T cells, and B cells) were purified separately from blood obtained from normal human donors. These cell types were cultured separately with 2 uM LXR-623 (or vehicle) for 18 hours, followed by RNA isolation and qPCR analysis for LXRα, LXRβ, ABCA1, and ABCG1. Without LXR-623 treatment, LXRα was found to be most highly expressed in monocytes, but expression of LXRα was also seen in T cells and B cells (Figure 6A). In contrast, basal expression levels of LXRβ were more similar in all cell types in PBMC (Figure 6B). Upon treatment with LXR-623, expression of LXRα mRNA was significantly increased in PBMC and monocytes, but not in T cells and B cells (Figure 6C), while LXRβ expression remained constant in all cell types regardless of LXR agonist treatment (Figure 6D). Interestingly, ABCA1 and ABCG1 differed in their regulation in different blood cell types following LXR agonist treatment. Monocytes were shown to express relatively high basal levels of ABCA1, and treatment with LXR-623 resulted in approximately 6 fold induction of ABCA1 mRNA levels (Figure 6E). T cells and B cells expressed very low, but measurable levels of ABCA1 mRNA, which was induced > 200 fold in T cells and > 20 fold in B cells, but the overall ABCA1 expression level in these cell types was still extremely low compared to PBMC and monocytes (Figure 6F). In contrast, ABCG1 was expressed and significantly regulated by LXR-623 in all PBMC cell types (Figure 6G).

### ABCA1 and ABCG1 expression is increased in peripheral blood of human subjects following oral administration of LXR-623

**Figure 7:**
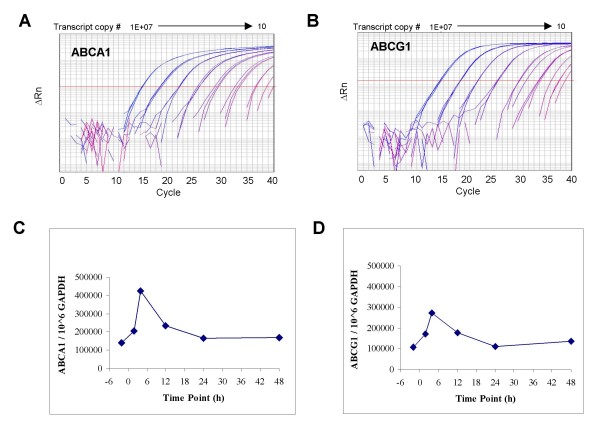
**ABCA1 and ABCG1 are upregulated in whole blood from human subjects following single-dose LXR-623**. Panels A and B. Ten-fold dilutions (ranging from 10 to 100,000,000 copies) of ABCA1 and ABCG1 *in vitro *transcripts were reverse transcribed into cDNA and PCR amplified on an ABI 7900 real time PCR system. Representative amplification plots are shown for ABCA1 (A) and ABCG1 (B) with each dilution analyzed in triplicate (the 10 copy dilution standards gave C_T_'s > 40 and are not shown on the graphs). For each analytical run, standard curves were generated from a dilution series of the calibrator transcripts to allow accurate copy number estimation in clinical RNA samples. Panels C and D: whole blood was collected into PAXgene tubes two hours prior to dosing (-2 h) and at 2 h, 4 h, 12 h, 24 h and 48 h following a single dose of LXR-623. Time course results for a single representative subject receiving 75 mg/kg LXR-623 are depicted for both ABCA1 (C) and ABCG1 (D) transcript levels. ABCA1 and ABCG1 transcript levels are expressed as actual copy numbers per million copies of GAPDH. Both transcripts exhibited similar fold elevations and followed identical time courses after LXR-623 exposure, with a maximal induction by 4 hours followed by a return to baseline levels after 24 hours.

In order to accurately and precisely measure ABCA1 and ABCG1 transcript levels in RNA from peripheral blood samples of human subjects prior to and following a single oral dose of LXR-623, external standard qRT-PCR assays for the two target genes and a normalizer transcript (GAPDH) were developed and analytically validated. Dilutions of *in vitro *ABCA1 and ABCG1 transcripts containing from 10 to 100,000,000 copies of ABCA1 and ABCG1 RNA were reverse transcribed into cDNA and PCR amplified on an ABI 7900 realtime PCR system (Figure 7, panels A and B). The interday efficiency for PCR amplification was 90.4% for ABCA1 (range: 84–100%) and 95.4% for ABCG1 (range: 90–104%). The calibration curves (n = 5 replicates at each level run on 5 separate days) for the ABCA1 and ABCG1 transcripts showed acceptable precision and accuracy (< 30% CV and 30% bias) from 1,000 to 100,000,000 copies. A similar external standard method was developed and analytically validated for the measurement of GAPDH RNA (data not shown). Normalized levels of ABCA1 mRNA ranged from 19,700 – 99,400 copies ABCA1/10^6 copies GAPDH in eleven healthy (untreated) subjects (6 males and 5 females, age 26-61 yrs), and levels of ABCG1 mRNA ranged from 34,500–104,600 copies ABCG1/10^6 copies GAPDH in the same subjects (data not shown).

Assessment of temporal profiles in each of the biomarker transcripts in peripheral blood collected from a representative subject receiving LXR-623 (Figure 7, panels C and D) revealed that peak transcriptional levels of ABCA1 and ABCG1 were detected 4 hours post-dosing, after which the levels of ABCA1 and ABCG1 decreased back to baseline levels by twenty-four to forty-eight hours. Strong dose-response and exposure-response relationships were observed for ABCA1 and ABCG1 transcriptional biomarkers in subjects receiving ascending doses of LXR-623, and these will be reported in a publication describing all of the results of the single ascending dose study in detail (A. Katz *et al*, submitted).

## Discussion

The intent of this work was to identify easily accessible, rapid, and robust indicators of LXR agonist exposure and activity to aid in the clinical development of synthetic LXR modulator compounds. An ideal surrogate tissue for such analyses is peripheral blood, but it was unclear whether LXR agonist activity could be monitored in peripheral blood. It was well known from the literature that activated macrophages (usually tissue-associated and not freely circulating in blood) respond to LXR agonists by increasing the expression of certain LXR target genes such as the ABC-cassette genes. Landis et al. [42] had previously reported that treatment of purified human primary monocytes in culture with a combination of oxidized LDL and 9-cis-retinoic caused the induction of TNFα expression and secretion, suggesting that LXRs may be expressed and functional in peripheral blood cells. But subsequent experiments to show that the monocytes' response to LXR agonist treatment was mediated by LXR binding to an LXR response element (LXRE) in the promoter of the TNFα gene were performed in cells transfected with an expression vector containing LXRα [42], so proof that circulating monocytes expressed functional LXRs was not conclusively established. There have been some reports of LXR expression and response to agonists in T-cells [43,44]. More recently, Siest et al [45] showed weak and variable expression of LXRα and LXRβ mRNA in PBMC from normal human donors using custom microarrays. However, this technique is relatively insensitive compared to qPCR, and no data were provided on the functionality of LXRs in PBMC. Therefore, we sought to determine whether transcriptional biomarkers of LXR activity could be monitored in peripheral blood.

Data presented here show that human peripheral blood mononuclear cells express LXRα and LXRβ. Surprisingly, functional LXR expression was found in T- and B-cells as well as in monocytes *ex vivo*. Evaluation of the transcriptional response of peripheral blood to synthetic LXR agonists *in vivo *was first performed in rats and mice, where expression of LXR target genes ABCA1 and ABCG1 was found to be significantly increased by different LXR agonist compounds, and as early as one hour following a single oral dose of LXR-623. These observations were then confirmed with experiments in higher species, in which monkeys given daily doses of LXR agonist compound showed robust and persistent expression changes in ABCA1 and ABCG1 in peripheral blood RNA after 7 days of dosing. These results were then extended to humans using blood cells from healthy subjects treated *ex vivo *with LXR-623. In both rats and humans given a single dose of LXR-623, the induction of ABCA1 and ABCG1 expression in peripheral blood cells tracked closely with plasma drug levels. Intriguingly, the elevation of ABCA1 and ABCG1 mRNA was not sustained beyond the peak of plasma LXR-623 concentration, suggesting a short *in vivo *t_1/2 _for these two mRNAs and the dependence of mRNA levels primarily upon transcription rate. This attribute is advantageous for pharmacodynamic biomarkers.

We applied global transcriptional profiling to human PBMC's treated with LXR-623 in culture to evaluate the repertoire of gene expression in peripheral blood and to determine whether the spectrum of transcriptional changes appeared to have biological relevance. It was found that many LXR target genes known to be regulated in macrophage, liver, or duodenum were also regulated in peripheral blood cells, and these genes were known to be involved in reverse cholesterol transport and lipid metabolism. This observation, combined with an apparent correlation between blood and spleen response to LXR agonists in the rat, suggests that the LXR response that can be monitored in peripheral blood may have clinical significance and might ultimately provide surrogate transcriptional markers of biological efficacy.

ABCA1 and ABCG1 were evaluated as pharmacodynamic markers of LXR-623 exposure in a single ascending dose study of LXR-623 in healthy human volunteers. In human whole blood RNA, ABCA1 and ABCG1 responded with similar temporal profiles following LXR-623 exposure in a representative human subject, indicating that the compound was appropriately engaging its target *in vivo *and eliciting the expected biological response. Future studies will attempt to correlate peripheral blood response to LXR agonist compound with ultimate biological efficacy endpoints.

## Conclusion

Peripheral blood cells show promise as a surrogate tissue for monitoring the activity of LXR modulator compounds in target organs. Several candidate biomarkers of LXR agonist exposure and activity have been identified in peripheral blood, and two of these (ABCA1 and ABCG1) have been demonstrated to change substantially (up to 200 fold change) and rapidly (≤ 4 hours) upon compound treatment. These transcriptional markers have been shown to be upregulated in peripheral blood cells from rodents, primates, and humans, and the magnitude of transcriptional induction of these biomarkers in peripheral blood cells closely corresponds to LXR agonist compound concentration in serum. These LXR biomarkers have already proven to be useful for the evaluation of a novel synthetic LXR agonist in a human clinical study.

## Competing interests

The authors declare that they have no competing interests.

## Authors' contributions

EAD, EMQ, MJE, MA, PN, MEB, AJD and ERL designed the experiments. EAD, EMQ, ARH, LC, IF, MDB, Q-YL, DS, SW, MA, TK, WMM, JAI, AMS, MEB, and ERL performed experiments and data analysis. EAD, EMQ, MJE, MA, MEB, AJD, and ERL provided data interpretation. ERL drafted the manuscript. All authors were consulted for critical evaluation of manuscript content, and all have given their approval to the final version of the manuscript.
